# Comparative efficacy and safety of pharmacological interventions for severe COVID-19 patients: An updated network meta-analysis of 48 randomized controlled trials

**DOI:** 10.1097/MD.0000000000030998

**Published:** 2022-10-14

**Authors:** Qinglin Cheng, Gang Zhao, Junfang Chen, Qingjun Jia, Zijian Fang

**Affiliations:** a Hangzhou Center for Disease Control and Prevention, Hangzhou, China; b School of Medicine, Hangzhou Normal University, Hangzhou, China.

**Keywords:** efficacy, network meta-analysis, randomized controlled trials, safety, severe COVID-19

## Abstract

To date, there has been little agreement on what drug is the "best" drug for treating severe COVID-19 patients. This study aimed to assess the efficacy and safety of different medications available at present for severe COVID-19.

**Methods:**

We searched databases for randomized controlled trials (RCTs) published up to February 28, 2022, with no language restrictions, of medications recommended for patients (aged 16 years or older) with severe COVID-19 infection. We extracted data on trials and patient characteristics, and the following primary outcomes: all-cause mortality (ACM), and treatment-emergent adverse events (TEAEs).

**Results:**

We identified 4021 abstracts and of these included 48 RCTs comprising 9147 participants through database searches and other sources. For decrease in ACM, we found that ivermectin/doxycycline, C-IVIG (i.e., a hyperimmune anti-COVID-19 intravenous immunoglobulin), methylprednisolone, interferon-beta/standard-of-care (SOC), interferon-beta-1b, convalescent plasma, remdesivir, lopinavir/ritonavir, immunoglobulin gamma, high dosage sarilumab (HS), auxora, and imatinib were effective when compared with placebo or SOC group. We found that colchicine and interferon-beta/SOC were only associated with the TEAEs of severe COVID-19 patients.

**Conclusion:**

This study suggested that ivermectin/doxycycline, C-IVIG, methylprednisolone, interferon-beta/SOC, interferon-beta-1b, convalescent plasma (CP), remdesivir, lopinavir/ritonavir, immunoglobulin gamma, HS, auxora, and imatinib were efficacious for treating severe COVID-19 patients. We found that most medications were safe in treating severe COVID-19. More large-scale RCTs are still needed to confirm the results of this study.

## 1. Introduction

To date, the World Health Organization has confirmed over 480 million cases.^[1]^ The mortality in patients with COVID-19 was estimated at 1.28%.^[1]^ For the current analyses, COVID-19 infection is the leading cause of the global burden of disease and public health, which has increased significantly since 2019, driven mainly by high morbidity, high impact, and mortality.^[2]^

Analysis of the data showed many pharmacologic interventions have been used to treat COVID-19 patients, which have been effective against COVID-19 infection.^[3–5]^ However, there were many compounds that differ in efficacy and safety, and it was not clear which drug was the “best” drug for treating severe COVID-19 patients. Due to the small sample size and time-updated lagged in previous meta-analyses and studies, there has been debate about the effectiveness and safety of drugs, with some findings contradicting each other for COVID-19 infection.^[6,7]^

Of primary concern was the medications on COVID-19 with all infection levels (i.e., mild, moderate and severe infection) in prior studies.^[8]^ Here, we had a critical shortage that we lacked an analysis stratified by different infection levels in medications of COVID-19. It is well known that the disease varies in infection, which could lead to individual treatment of different infection subgroups in patients.^[9]^

Though the network meta analysis (NMA) has been performed in prior reports,^[7]^ there was little published data on the network study for the therapy of severe COVID-19 patients. Additionally, we found that there have been further randomized controlled trials (RCTs) of some other drugs for the treatment of COVID-19 through the literature search.^[10]^ Some large-scale RCTs have been completed in the treatment of COVID-19 infection. We urgently need an updated assessment of the available evidence to support clinical decision-making.

How do we select pharmacological interventions for severe COVID-19 patients in clinical practice? In order to fill this gap, we did an updated NMA of RCTs for drug interventions of severe COVID-19 infection. The purpose of this study is to evaluate the efficacy and safety of medications based on RCTs of the available evidence for severe COVID-19 infection.

## 2. Materials and Methods

### 2.1. Protocol and registration

We performed the present NMA in accordance with the guidelines of the Preferred Reporting Items for Systematic Reviews and Meta-Analyses statement.^[11]^ This study was a review article and did not involve a research protocol requiring approval by a relevant institutional review board or ethics committee. Informed consent was also not applicable. We registered the protocol in the International Prospective Register of Systematic Reviews (PROSPERO) (CRD42021293879).

### 2.2. Search strategy and selection criteria

We conducted a systematic literature search in 8 electronic databases (PubMed, Elsevier Science Direct, Cochrane Library, Google Scholar, Springer Link, MedRxiv, China National Knowledge Infrastructure, and Wanfangdata) for RCTs published, with no language restrictions from their inception to February 28, 2022. We included the RCTs on the treatment of severe COVID-19 patients (aged 16 years or older). The appendix had full search strategies listed (Supplemental Appendix 1, http://links.lww.com/MD/H533). We extracted data on RCTs, patient and medications characteristics (Table 1).

**Table 1:** Characteristics of randomized controlled trials of pharmacological interventions in severe patients with COVID-19.

Author name (reference)	Publication yr	Country/Countries of origin	Study design	Method of COVID-19 testing	Numbers of participants	Gender*	Age (yrs)	Interventions	Treatment medication dose	Controls	Control medication dose	Follow-up time (d)	Primary outcomes
Absalón-Aguilar A et al ^[18]^	2021	Mexico	TRPCTs	RT-PCR	116	Males (n = 76); Females (n = 40)	≥ 18	Colchicine (n = 56)	Patients received 1.5 mg of colchicine at baseline, which corresponded to the day of the patient’s recruitment in the study, and, then, 0.5 mg PO BID for 10 days	Placebo (n = 60)	Patients received placebo PO at baseline, which corresponded to the day of the patient’s recruitment in the study, and, then, 0.5 mg PO BID for 10 days	10	ACM; ratio of TEAEs
Ader F et al ^[19]^	2022	Europe	MDRCTs	RT-PCR	328	Not specified	≥ 18	Remdesivir/SOC (n = 161)	Remdesivir was administered intravenously at a loading dose of 200 mg on day 1 followed by a 100 mg, 1-h infusion once-daily for a total duration of 10 days	SOC (n = 167)	Lopinavir–ritonavir (LPV/R), LPV/R and interferon beta-1a, or hydroxychloroquine	29	ACM
Ali S et al ^[20]^	2021	Pakistan	RCTs	RT-PCR	50	Males (n = 35); Females (n = 15)	≥ 18	Hyperimmune anti-COVID-19 Intravenous Immunoglobulin (C-IVIG) (n = 40)	5% C-IVIG dosage arms (0.15, 0.20, 0.25, 0.30 g/kg)	SOC (n = 10)	Not specified	28	ACM; ratio of TEAEs
AlQahtani Met al.^[21]^	2021	Bahrain	RCTs	RT-PCR	40	Males (n = 32); Females (n = 8)	≥ 21	Convalescent plasma (n = 20)	The dosage of CP was 400 mL, given as 200 mL over 2 h over 2 successive days	SOC (n = 20)	Possible therapy including antiviral medications, tocilizumab and antibacterial medication	28	ACM; ratio of TEAEs
AlShehry Net al. ^[22]^	2021	Saudi Arabia	RCTs	RT-PCR	164	Males (n = 137); Females (n = 27)	≥ 18	Convalescent plasma/SOC (n = 40)	300 mL (200–400 mL/treatment dose) convalescent plasma at least once, and if required, daily for up to 5 sessions	SOC (n = 124)	Not specified	30	ACM
Aman Jet al. ^[10]^	2021	Netherlands	DRPCTs	RT-PCR	385	Males (n = 264); Females (n = 121)	≥ 18	Imatinib (n = 197)	800 mg on day 0 followed by 400 mg daily on d 1–9	Placebo (n = 188)	Not specified	28	ACM
Avendano Sola C et al ^[23]^	2020	Spain	MRCTs	RT-PCR	81	Males (n = 44); Females (n = 37)	≥ 18	Convalescent plasma (n = 38)	Receive 1 dose (250-300 mL) of convalescent plasma	SOC (n = 43)	Not specified	97	ACM; ratio of TEAEs
Beltran-Gonzalez JLet al. ^[24]^	2021	Mexico	DRPCTs	RT-PCR	106	Males (n = 66); Females (n = 40)	53 ± 16.9	Hydroxychloroquine (n = 33)	Hydroxychloroquine 400 mg every 12 h on the first day and subsequently, 200 mg every 12 h for 4 days	Placebo (n = 37)	Not specified	30	ACM
Beltran-Gonzalez JL et al ^[24]^	2021	Mexico	DRPCTs	RT-PCR	106	Males (n = 66); Females (n = 40)	53 ± 16.9	Ivermectin (n = 36)	Ivermectin, 12 mg or 18 mg	Placebo (n = 37)	Not specified	30	ACM
Bruen Cet al^[25]^	2022	USA	DRPCTs	RT-PCR	261	Males (n = 176); Females (n = 85)	≥ 18	Auxora/SOC (n = 130)	Auxora was administered by a 4-h IV infusion at 2.0 mg/kg (1.25 mL/kg) at 0-h and 1.6 mg/kg (1 mL/kg) at 24 and 48 h	Placebo/SOC (n = 131)	Placebo was dosed at 1.25 mL/kg at 0-h and 1 mL/kg at 24 and 48 h	60	ACM
Cao Bet al.^[26]^	2020	China	RCTs	RT-PCR	199	Males (n = 120); Females (n = 69)	≥ 18	Lopinavir/ritonavir (n = 99)	Lopinavir–ritonavir (400 mg and 100 mg: respectively) twice a day for 14 days	SOC (n = 100)	Not specified	28	ACM; ratio of TEAEs
Cao Yet al.^[27]^	2020	China	RPCTs	RT-PCR	41	Males (n = 24); Females (n = 17)	≥ 18	Ruxolitinib/SOC (n = 20)	5 mg twice a day	Placebo (n = 21)	Not specified	28	ACM; ratio of TEAEs
Caricchio R et al ^[28]^	2021	Europe and the United States	DRPCTs	RT-PCR	454	Males (n = 267); Females (n = 187)	≥ 18	Canakinumab/SOC (n = 225)	A single intravenous infusion of canakinumab (450 mg for body weight of 40-<60 kg, 600 mg for 60-80 kg, and 750 mg for > 80 kg; n = 227)	Placebo/SOC (n = 223)	Not specified	29	ACM; ratio of TEAEs
Cremer PCet al.^[29]^	2021	USA	MDRPCTs	RT-PCR	40	Males (n = 26); Females (n = 14)	≥ 18	Mavrilimumab (n = 21)	Receive mavrilimumab 6 mg/kg as a single intravenous infusion	Placebo (n = 19)	Not specified	28	ACM; ratio of TEAEs
Davoudi-Monfared Eet al.^[30]^	2020	Iran	RCTs	RT-PCR	81	Males (n = 44); Females (n = 37)	≥ 18	Interferon-beta-1a/SOC (n = 42)	44 mg/mL (12 million IU/mL) dose of interferon-beta-1a 3 times weekly for 2 consecutive weeks	SOC (n = 39)	HCQ (400 mg twice a day [BID] on the first day and then 200 mg BD) plus LPV/r (400 and 100 mg: respectively: BD) or atazanavir-ritonavir (300 and 100 mg: respectively: daily) for 7–10 days	28	ACM
de Alencar JCGet al. ^[31]^	2021	Brazil	DRPCTs	RT-PCR	135	Males (n = 80); Females (n = 55)	≥ 18	N-acetylcysteine (NAC, n = 67)	N-acetylcysteine (NAC,28 mg/mL and 14 mg/mL)	Placebo (n = 68)	Dextrose 5% in water (1000 mL in total) intravenously	9	ACM
Dequin PFet al.^[32]^	2020	France	DRPCTs	RT-PCR	149	Males (n = 104); Females (n = 45)	≥ 18	Hydrocortisone (n = 76)	200 mg/d until day 7, 100 mg/d for 4 days and 50 mg/d for 3 days, for a total of 14 days	Placebo (n = 73)	Not specified	14	ACM
Edalatifard M et al ^[33]^	2020	Iran	RCTs	RT-PCR	62	Males (n = 39); Females (n = 23)	≥ 18	Methylprednisolone/SOC (n = 34)	Intravenous injection: 250 mg per day for 3 days	SOC (n = 28)	HCQ sulfate: lopinavir and naproxen	48	ACM
Ely EWet al^[34]^	2022	Argentina, Brazil, Mexico, and the USA	MDRPCTs	RT-PCR	101	Males (n = 55); Females (n = 46)	≥ 18	Baricitinib/SOC (n = 51)	Baricitinib 4 mg plus SOC was crushed for nasogastric tube delivery (or given orally when feasible) and given once daily for up to 14 days or until discharge from hospital, whichever occurred first	Placebo/SOC (n = 50)	Matched placebo plus SOC	60	ACM; ratio of TEAEs
Furtado RHMet al.^[35]^	2020	Brazil	MDRCTs	RT-PCR	397	Males (n = 262); Females (n = 135)	≥ 18	Azithromycin/SOC (n = 214)	500 mg azithromycin once daily plus SOC for 10 days	SOC (n = 183)	SOC without macrolides: at the discretion of treating hysicians and according to local guidelines	29	ACM
Gharebaghi Net al.^[36]^	2020	Iran	DRPCTs	RT-PCR	59	Males (n = 41); Females (n = 18)	≥ 18	Immunoglobulin gamma (n = 30)	IVIg (human) flebogamma 5% DIF GRIFOLS daily for 3 consecutive days	Placebo (n = 29)	Not specified	28	ACM
Hashim HAet al.^[37]^	2021	Iraq	RCTs	RT-PCR	140	Males (n = 73); Females (n = 67)	16 to 86	Ivermectin/doxycycline (n = 70)	Treated with 200ug/kg PO of Ivermectin per day for 2-3 days along with 100 mg PO doxycycline twice per day for 5-10 days	SOC (n = 70)	Not specified	28	ACM
Hernandez-Cardenas Cet al. ^[38]^	2021	Mexico	DRPCTs	RT-PCR	214	Males (n = 161); Females (n = 53)	≥ 18	Hydroxychloroquine (n = 106)	200 mg every 12 h, for 10 days	Placebo (n = 108)	Not specified	30	ACM; ratio of TEAEs
Krolewiecki Aet al. ^[39]^	2021	Argentina	RCTs	RT-PCR	32	Males (n = 17); Females (n = 15)	≥ 18	Ivermectin (n = 20)	Patients in the ivermectin group received oral treatment for 5 consecutive days with either breakfast or lunch at approximately 24 h intervals. Ivermectin 6 mg ranurated tablets (IVER P, Laboratorios Elea/Phoenix, and Argentina) were used in all cases at a dose of 600 mg/kg/day based on baseline weight rounding to the lower full (6 mg) and half (3 mg) dose.	SOC (n = 12)	Not specified	30	Ratio of TEAEs
Lescure FXet al.^[40]^	2021	Argentina, Brazil, Canada, Chile, France, Germany, Israel, Italy, Japan, Russia, and Spain	MDRPCTs	RT-PCR	416	Males (n = 261); Females (n = 155)	≥ 18	Sarilumab 200 mg (n = 159)	Intravenous sarilumab 200 mg	Placebo (n = 84)	Not specified	60	ACM; ratio of TEAEs
Lescure FXet al.^[40]^	2021	Argentina, Brazil, Canada, Chile, France, Germany, Israel, Italy, Japan, Russia, and Spain	MDRPCTs	RT-PCR	416	Males (n = 261); Females (n = 155)	≥ 18	Sarilumab 400 mg (n = 173)	Intravenous sarilumab 400 mg	Placebo (n = 84)	Not specified	60	ACM; ratio of TEAEs
Li Let al.^[41]^	2020	China	MRCTs	RT-PCR	103	Males (n = 60); Females (n = 43)	≥ 18	Convalescent plasma/SOC (n = 52)	The dose of convalescent plasma was approximately 4 to 13 mL/kg of recipient body weight.	SOC (n = 51)	Not specified	74	ACM
Libster Ret al.^[42]^	2021	Argentina	DRPCTs	RT-PCR	160	Males (n = 60); Females (n = 100)	≥ 65	Convalescent plasma (n = 80)	Convalescent plasma 250 mL with an IgG titer greater than 1:1000	Placebo (n = 80)	Not specified	25	ACM
Miller Jet al.^[43]^	2020	American	RCTs	RT-PCR	30	Males (n = 14); Females (n = 16)	≥ 18	Auxora (n = 20)	Auxora was administered on 3 consecutive days as a 4-h continuous intravenous infusion. The initial dose was 2.0mg/kg (max 250 mg): and subsequent doses were 1.6 mg/kg (max 200 mg) at 24 and 48 h	SOC (n = 10)	Not specified	30	ACM
Munch MWet al.^[44]^	2021	Denmark, Sweden, Switzerland and India	MDRPCTs	RT-PCR	30	Males (n = 24); Females (n = 6)	≥ 18	Hydrocortisone (n = 16)	Hydrocortisone 200mg per day for 7days or until discharge	Placebo (n = 14)	Not specified	90	ACM
Olender SAet al.^[45]^	2020	United States	RCTs	RT-PCR	1114	Males (n = 672); Females (n = 442)	≥ 18	Remdesivir (n = 298)	Remdesivir 200 mg on day 1: followed by remdesivir 100 mg daily on days 2–5; or SOC plus remdesivir 200 mg on day 1: followed by remdesivir 100 mg daily on days 2–10	SOC (n = 816)	Allowed to receive medications that may potentially treat COVID-19: excluding remdesivir	30	ACM
Patel Jet al.^[46]^	2021	UK	DRPCTs	RT-PCR	806	Males (n = 577); Females (n = 229)	18 to 79	Otilimab (human anti-GM-CSF monoclonal) (n = 403)	A single dose of otilimab resulted in mean Cmax of 20.2 μg/mL after dosing on Day 1 and 1.9 μg/mL on Day 7	Placebo (n = 4 03)	Not specified	28	ACM
Rahmani Het al. ^[47]^	2020	Iran	RCTs	RT-PCR	66	Males (n = 39); Females (n = 27)	≥ 18	Interferon-beta-1b (n = 33)	Patients in the interferon group received interferon-beta-1b (250 mcg subcutaneously every other day for 2 consecutive weeks) along with the national protocol medications	SOC (n = 33)	Patients received only the national protocol medications (lopinavir/ritonavir or atazanavir/ritonavir plus hydroxychloroquine for 7–10 days	28	ACM
Rasheed AMet al. ^[48]^	2020	Iraq	RCTs	RT-PCR	49	Not specified	≥ 18	Convalescent plasma (n = 21)	Not specified	SOC (n = 28)	Not specified	60	ACM; ratio of TEAEs
Rea-Neto Aet al.^[49]^	2021	Brazil	RCTs	RT-PCR	105	Males (n = 70); Females (n = 35)	≥ 18	Chloroquine (CQ)/hydroxychloroquine (HCQ) (n = 53)	CQ 450 mg BID on day 1 and 450 mg once daily from day 2 to 5 and HCQ 400 mg BID on day 1 and 400 mg once daily from day 2 to 5	SOC (n = 52)	Not specified	28	ACM
Rea-Neto A et al^[49]^	2021	Brazil	RCTs	RT-PCR	105	Males (n = 70); Females (n = 35)	≥ 18	Chloroquine (CQ) (n = 24)	CQ 450 mg BID on day 1 and 450 mg once daily from day 2 to 5	SOC (n = 52)	Not specified	28	ACM
Rea-Neto Aet al.^[49]^	2021	Brazil	RCTs	RT-PCR	105	Males (n = 70); Females (n = 35)	≥ 18	Hydroxychloroquine (HCQ) (n = 29)	HCQ 400 mg BID on day 1 and 400 mg once daily from day 2 to 5	SOC (n = 52)	Not specified	28	ACM
Rosas IOet al.^[50]^	2021	Europe and North America	MDRPCTs	RT-PCR	438	Males (n = 306); Females (n = 132)	≥ 18	Tocilizumab (n = 294)	8 mg per kilogram of body weight, with a maximum dose of 800 mg	Placebo (n = 144)	Not specified	28	ACM; ratio of TEAEs
Sehgal ISet al.^[51]^	2021	India	DRPCTs	RT-PCR	42	Males (n = 29); Females (n = 13)	≥ 18	Mycobacterium-w (n = 20)	Each dose of 0.1 mL Mw contains 0.5 × 109 heat killed Mycobacterium w, 0.9% sodium chloride, and 0.01% thimerosal (as preservative)	Placebo (n = 22)	0.9% sodium chloride, 0.01% thiomersal	28	ACM; ratio of TEAEs
Shi Let al. ^[52]^	2021	China	DRPCTs	RT-PCR	100	Males (n = 56); Females (n = 44)	≥ 18	Human umbilical cord-derived mesenchymal stem cells (UC-MSCs) (n = 65)	UC-MSCs (4 × 107 cells per infusion)	Placebo (n = 35)	Not specified	28	Ratio of TEAEs
Simonovich VAet al. ^[53]^	2020	Argentina	RPCTs	RT-PCR	333	Males (n = 225); Females (n = 108)	≥ 18	Convalescent plasma (n = 228)	Received convalescent plasma	Placebo (n = 105)	Not specified	30	ACM; ratio of TEAEs
Sivapalasingam Set al. ^[54]^	2021	USA	DRPCTs	RT-PCR	126	Males (n = 531); Females (n = 219)	≥ 18	Sarilumab 200 mg group (n = 50)	Intravenous sarilumab 200 mg	Placebo (n = 25)	Not specified	22	ACM; ratio of TEAEs
Sivapalasingam Set al. ^[54]^	2021	USA	DRPCTs	RT-PCR	126	Males (n = 531); Females (n = 219)	≥ 18	Sarilumab 400 mg group (n = 51)	Intravenous sarilumab 400 mg	Placebo (n = 25)	Not specified	22	ACM; ratio of TEAEs
Solanich Xet al.^[55]^	2021	Spain	RCTs	RT-PCR	55	Males (n = 23); Females (n = 21)	≥ 18	Methylprednisolone pulses (n = 27)	methylprednisolone pulses of 120 mg/day had to be administered on 3 consecutive days	SOC (n = 28)	Not specified	56	ACM
Temesgen Z et al ^[56]^	2021	USA and Brazil	DRPCTs	RT-PCR	479	Males (n = 310); Females (n = 169)	≥ 18	Lenzilumab (n = 236)	Three doses of lenzilumab (600 mg, each) or placebo were administered 8 hours apart via a 1-hour IV infusion	Placebo (n = 243)	Not specified	28	ACM; ratio of TEAEs
Veiga VCet al.^[57]^	2021	Portugal	DRCTs	RT-PCR	129	Males(n = 88); Females(n = 41)	≥ 18	Tocilizumab/SOC (n = 65)	Single intravenous infusion of 8 mg/kg	SOC (n = 64)	Not specified	15	ACM
Zhang Jet al. ^[58]^	2021	China	RCTs	RT-PCR	56	Males (n = 36); Females(n = 20)	18 to 80	High-dose intravenous vitamin C (HDIVC) (n = 27)	50 mL every 12 h for 7 days at a rate of 12 mL/hour	Placebo (n = 29)	Bacteriostatic water for injection (50 mL every 12 h for 7 days at a rate of 12 ml/hour)	28	ACM
Zhong Met al.^[59]^	2020	China	RPCTs	RT-PCR	17	Males (n = 13); Females (n = 4)	51 to 91	α-Lipoic acid (n = 8)	1200 mg/d, intravenous infusion	Placebo (n = 9)	Equal volume saline infusion (placebo) for 7 days	30	ACM

ACM = all-cause mortality, DRPCTs = double-blind, randomized placebo-controlled trials, COVID-19 = coronavirus disease 2019, MDRCTs = multicenter, double-blind, randomized controlled trials, MDRPCTs = multicenter, double-blind, randomized placebo-controlled trials, MRCTs = multicenter, randomized controlled trials, RCTs = randomized controlled trials, RPCTs = randomized placebo-controlled trials, RT-PCR = reverse transcription-polymerase chain reaction, SOC = standard of care, TEAEs = treatment-emergent adverse events, TRPCTs = three-blind, randomized placebo-controlled trials, UC-MSCs = human umbilical cord-derived mesenchymal stem cells.

* If the total number of gender is not equal to the number of participants, it is due to loss to follow-up from participants.

To determine their eligibility, we reviewed the abstracts and full-texts of potentially relevant articles. We selected articles for the evaluation based on the criterion: at least one statistical analysis of the association between severe COVID-19 and medications was presented and described as an assessment for efficacy or safety.

All RCTs that measured the efficacy or safety between drug interventions and severe COVID-19 infection were considered for inclusion. We listed full inclusion and exclusion criteria in the appendix (Supplemental Appendix 2, http://links.lww.com/MD/H534). During the selection of qualified studies, we resolved any ambiguity through mutual discussion and consensus.

### 2.3. Data extraction

At least 2 independent investigators (JQJ, CJF, and FZJ) extracted and entered onto all data through a standardized data extraction form. The main data extracted was the assessments of efficacy and safety. We collected the following information: basic characteristics, including author name, publication year, country/countries of origin, study design, method of COVID-19 testing, patient population, age, gender, sample size, interventions, treatment medication dose, controls, control medication dose, follow-up time, and primary outcomes; Primary outcomes, including all-cause mortality (ACM) and rate of treatment-emergent adverse events (TEAEs). We also contacted the authors if they did not report the above data information in the published article. One independent investigator undertook a preliminary extraction of studies, and another investigator reviewed the extraction. Differences were discussed, and a third researcher participated (CQL) if they reached no agreement.

### 2.4. Quality assessment

At least 2 investigators (ZG, CQL, JQJ, CJF, and FZJ) evaluated the risk of bias for all studies. We estimated the risk of bias with the Cochrane Risk-of-Bias Tool.^[12]^ We assessed the certainty of evidence by using the Grading of Recommendations Assessment, Development, and Evaluation approach for the NMA.^[13]^

### 2.5. Outcome measures and definitions

The severe COVID-19 infection represented patients with fever or suspected respiratory infection, plus one of the following: respiratory rate > 30 breaths/min, severe respiratory distress, or SpO2 ≤ 93% on room air.^[14,15]^ The primary outcomes were the ACM and TEAEs for severe COVID-19 patients, from the beginning of treatment to the end of follow-up. The definitions of ACM, TEAEs, and severe COVID-19 patients can refer to our previous study.^[14]^

### 2.6. Data analysis

#### 2.6.1. Assessment of the transitivity assumption.

Transitivity is the fundamental assumption of indirect comparisons and NMA. We investigated the distribution of potential effect modifiers to assess the transitivity assumption. Potential effect modifiers included multicenter study, duration of study (DS), blinding, crossover design, sample size (SS), industry sponsorship, and risk of reported bias (RRB).

#### 2.6.2. Network meta-analysis.

We used STATA statistical software (Version 15, Stata Corporation, and College Station, Texas, USA) to conduct NMAs. We analyzed binary variables (ACM and TEAEs) using odds ratio (OR) with 95% confidence interval (CI). The appendix reported additional details (Supplemental Appendix 3, http://links.lww.com/MD/H535). We defined statistical significance as the *P* value < .05.

We merged simultaneously the direct evidence and indirect evidence or different indirect evidence through an NMA. In NMA, we analyzed the effect of intervention using group-level data. A fixed-effect model (i.e., *I*^2^ ≤ 50%) or a random-effect model (i.e., *I*^2^ > 50%) was used to summarize the effect sizes of NMA. We ranked the therapeutic effect for each outcome using the surface under the cumulative ranking area (SUCRA) curve and mean ranks.^[16]^ In addition, the endpoints which lower was better would show rank 1 was the best and rank N was the worst based on figures and vice versa.

#### 2.6.3. Assessment of heterogeneity and inconsistency.

We used the node splitting method (i.e., split evidence on a specific comparison into direct and indirect evidence) to estimate the inconsistency of NMAs.^[17]^ There was no significant inconsistency in outcomes if *P* > .05.

#### 2.6.4. Assessment of the risk of bias.

We assessed the risk of bias of included studies using the Cochrane Collaboration’s Tool for Assessing Risk of Bias,^[12]^ classifying the risk of bias as high, unclear, or low. We evaluated the small-study effect using comparison adjusted funnel plots.^[17]^

#### 2.6.5. Sensitivity analysis.

The inclusion of various study designs and populations might contribute to heterogeneity and inconsistency. We used sensitivity analysis to evaluate the effect of our conclusions. We analyzed the data using the following restrictions: multicenter study, DS, blinding, crossover design, SS, industry sponsorship, and RRB.

We used the “netmeta” package in R (version 4.0.5) to duplicate the NMAs of primary outcomes.

## 3. Results

### 3.1. Description of included studies

**Figure 1.**
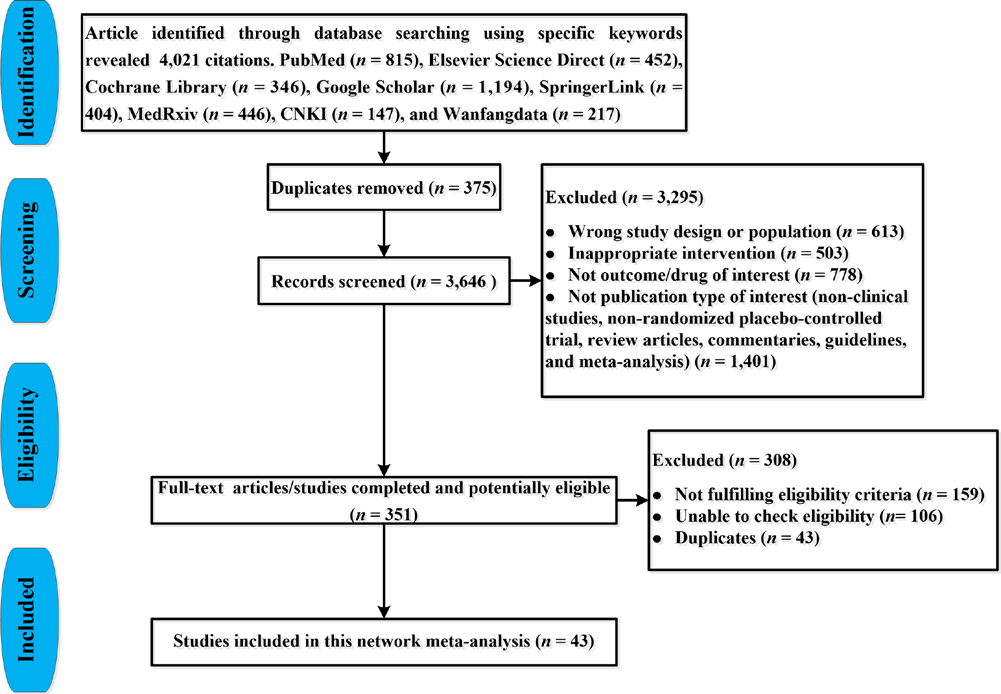
PRISMA flow-chart for study selection.

The search identified 4021 citations, out of which 3670 were excluded for duplications, wrong study design or population (i.e., non-severe COVID-19 infection), inappropriate intervention, not outcome/drug of interest, non-clinical studies, non-RCTs, review articles, commentaries, guidelines, and meta-analysis by checking titles and abstracts. The full texts of 351 articles were obtained to check eligibility, in which we excluded 308 articles for non- fulfilling eligibility criteria, unable to check eligibility and duplications. Finally, 43 studies^[10,18–59]^ were included in our NMA. Figure 1 shows the selection process for the included studies.

Forty-eight RCTs, including 9147 patients, were included (Fig. 1) and described in Table 1. This analysis had a mean sample size of 106 [interquartile range 58–238]. The age of subjects was greater than or equal to 16 years. The median duration of follow-up treatment was 28 days (interquartile range 27–30).

### 3.2. Quality appraisal

**Figure 2.**
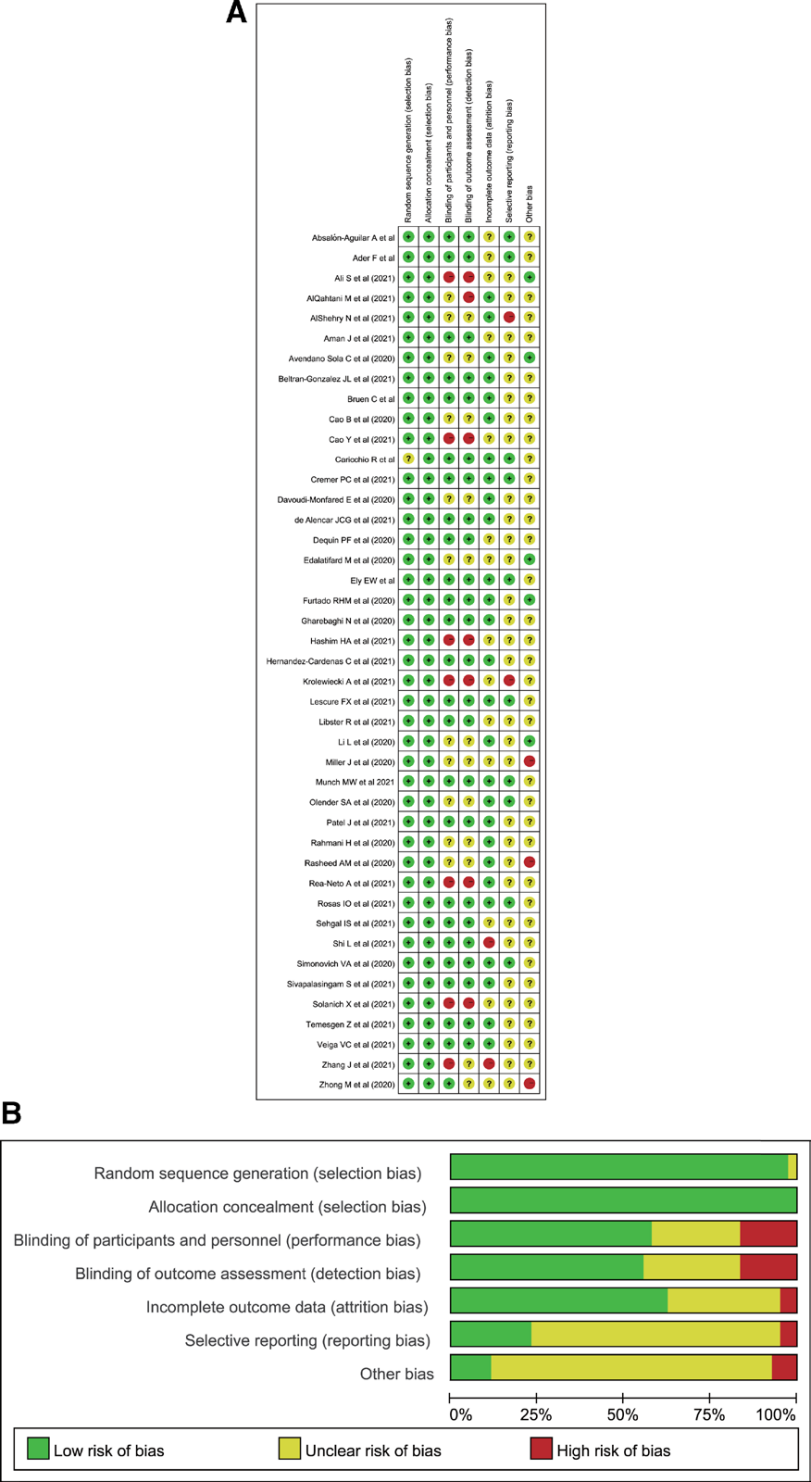
The quality of the included randomized controlled trials. (A) Risk of bias summary (Note: The yellow circle with question mark represents “unclear risk of bias”, the red one with minus sign represents “high risk of bias” and the green one with plus sign represents “low risk of bias”). (B) Risk of bias graph.

The included RCTs were of good quality (Fig. 2). Figure 2 also showed the risk of bias summary.

### 3.3. Network of evidence

**Figure 3.**
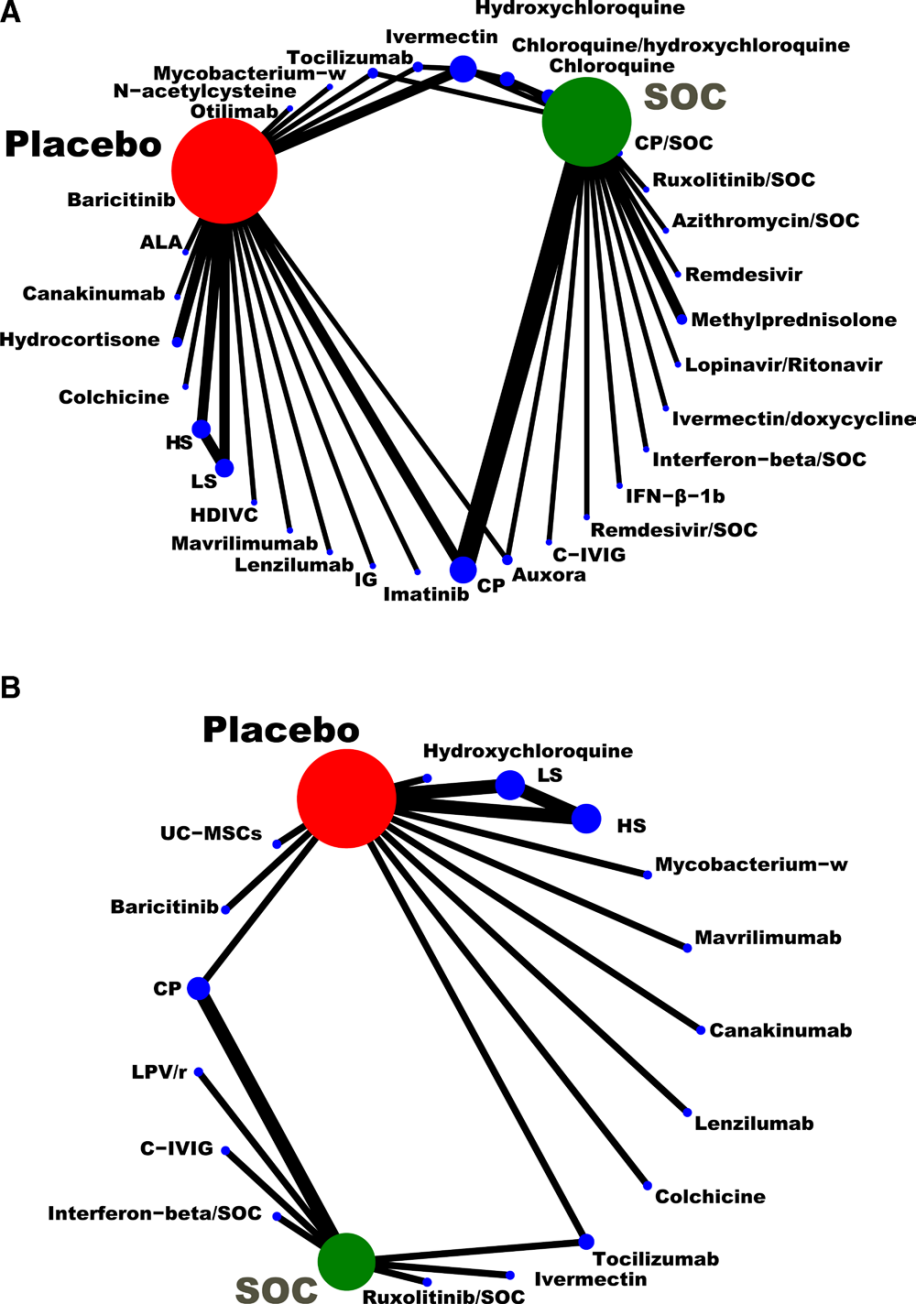
Network plot of eligible comparisons for all-cause mortality (A), and the ratio of treatment-emergent adverse events (B) for medications in patients with severe COVID-19. ALA= α-Lipoic acid, C-IVIG = hyperimmune anti-COVID-19 intravenous immunoglobulin, COVID-19 = coronavirus disease 2019, CP = convalescent plasma, IFN-β= interferon-beta, IG = immunoglobulin gamma, HDIVC = high-dose intravenous vitamin C, HS = high dosage sarilumab, LS = low dosage sarilumab, SOC = standard-of-care, UC-MSCs = human umbilical cord-derived mesenchymal stem cells.

In the network of connected RCTs (Fig. 3), the width of the lines corresponded to the number of trials included each treatment comparison. From Figure 3, we could see that the result was well connected. As shown in Figure 3A, the standard of care (SOC) was the most well-connected treatment, with chloroquine (CQ), hydroxychloroquine (HCQ), CQ/HCQ, convalescent plasma (CP), CP/SOC, remdesivir, remdesivir/SOC, lopinavir/ritonavir (LPV/r), interferon-beta-1b (IFN-β-1b), C-IVIG (i.e., a hyperimmune anti-COVID-19 intravenous immunoglobulin), ivermectin/doxycycline, IFN-β/SOC, tocilizumab, ruxolitinib/SOC, methylprednisolone, azithromycin/SOC, and auxora directly connected to it. Sarilumab such as high dosage sarilumab (HS) and low dosage sarilumab (LS), CQ/HCQ, ivermectin, canakinumab, colchicine, baricitinib, immunoglobulin gamma (IG), α-Lipoic acid, imatinib, mavrilimumab, lenzilumab, mycobacterium-w, otilimab, N-acetylcysteine, tocilizumab, high-dose intravenous vitamin C, hydrocortisone, and CP were directly connected to placebo in this network plot. Several sources of indirect evidence were available to inform comparisons between ivermectin, tocilizumab, HCQ, CP, auxora, placebo, and SOC (Fig. 3A). In Figure 3B, there was also a direct connection between SOC and C-IVIG, CP, LPV/r, ruxolitinib/SOC, ivermectin, IFN-β/SOC, and tocilizumab, or between placebo and HS, LS, mycobacterium-w, mavrilimumab, colchicine, canakinumab, baricitinib, human umbilical cord-derived mesenchymal stem cells, HCQ, lenzilumab, CP or tocilizumab. Several indirect connections were available between SOC and CP, tocilizumab, placebo (Fig. 3B).

### 3.4. Efficacy outcomes

**Figure 4.**
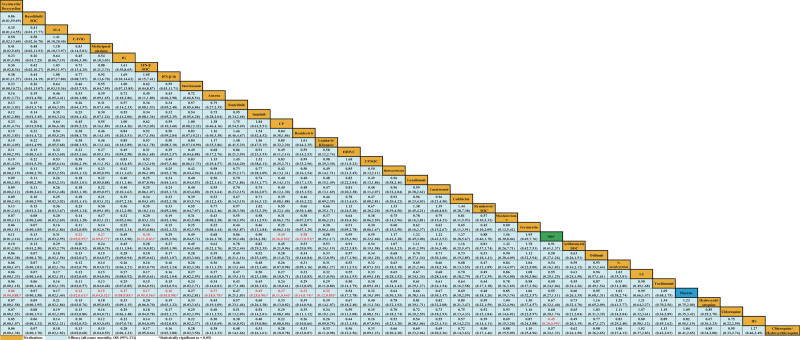
Network meta-analyses of the relative efficacy and safety of medications for all-cause mortality among patients with severe COVID-19 infection. The red font and asterisk indicated comparisons that were statistically significant. ALA= α-Lipoic acid, C-IVIG = hyperimmune anti-COVID-19 intravenous immunoglobulin, CI = confidence interval, COVID-19 = coronavirus disease 2019, CP = convalescent plasma, HDIVC = high-dose intravenous vitamin C, HS = high dosage sarilumab, IFN-β=interferon-beta, IG = immunoglobulin gamma, LS = low dosage sarilumab, OR = odds ratio, SOC = standard-of-care.

Forty-one studies^[10,18–38,40–51,53–59]^ reported ACM as outcome measurement (Supplemental Table S1, http://links.lww.com/MD/H536). We found that C-IVIG (OR 0.22, 95% CI 0.05-0.95), methylprednisolone (OR 0.27, 95% CI 0.09-0.77), IFN-β/SOC (OR 0.30, 95% CI 0.11-0.83), CP (OR 0.49, 95% CI 0.26-0.94), remdesivir (OR 0.58, 95% CI 0.37-0.93), and HS (OR 0.45, 95% CI 0.20-0.99) were associated with the decrease of ACM when compared with the SOC group (Fig. 4). Unfortunately, there was no significant difference in other 27 medications (e.g., ivermectin/doxycycline, IFN-β-1b, LPV/r, IG, LPV/r, and imatinib) or placebo for ACM when compared with SOC.

For a decrease in ACM, ivermectin/doxycycline (OR 0.06, 95% CI 0.00-0.88), C-IVIG (OR 0.12, 95% CI 0.02-0.61), methylprednisolone (OR 0.15, 95% CI 0.04-0.52), IG (OR 0.27, 95% CI 0.08-0.85), IFN-β/SOC (OR 0.17, 95% CI 0.05-0.56), IFN-β-1b (OR 0.16, 95% CI 0.03-0.98), auxora (OR 0.37, 95% CI 0.18-0.78), imatinib (OR 0.49, 95% CI 0.25-0.96), CP (OR 0.27, 95% CI 0.11-0.64), remdesivir (OR 0.32, 95% CI 0.14-0.74), and LPV/r (OR 0.32, 95% CI 0.12-0.85) were effective when compared with the placebo group (Fig. 4). We did not identify that there was a difference between placebo and other 22 medications (e.g., ruxolitinib/SOC, ivermectin, tocilizumab, HS, and mavrilimumab) or SOC for the ACM of severe COVID-19 infection (Fig. 4).

The Supplemental Figure S1, http://links.lww.com/MD/H538 presented the ranking for the ACM of medications in severe COVID-19 patients based on cumulative probability plots and SUCRA.

### 3.5. Safety outcomes

**Figure 5.**
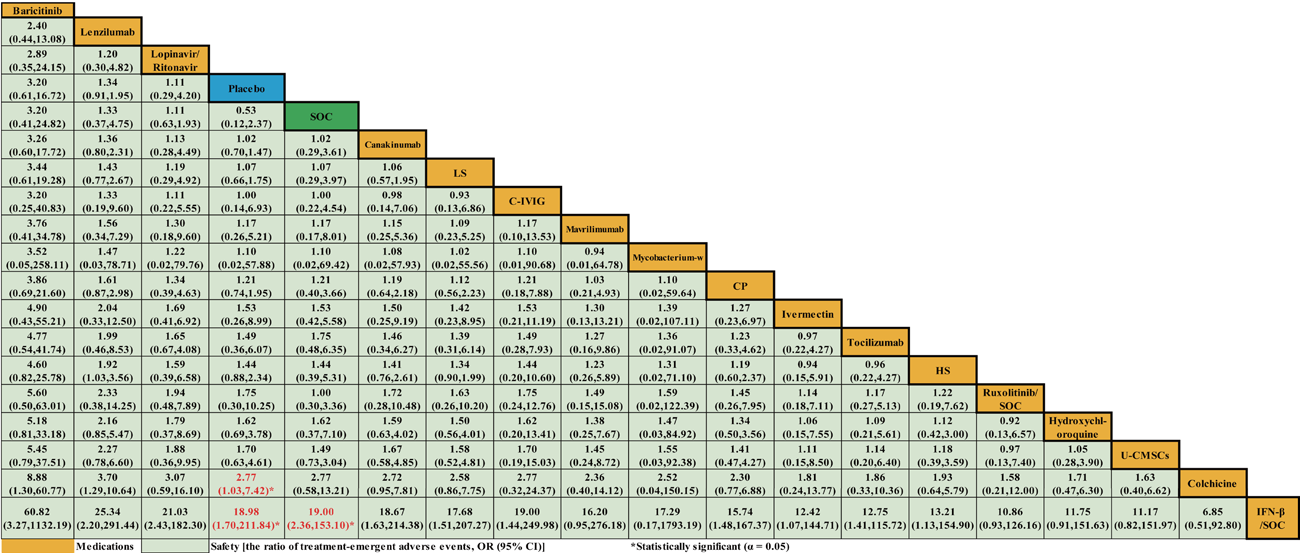
Network meta-analyses of the relative efficacy and safety of medications for the ratio of treatment-emergent adverse events among patients with severe COVID-19 infection. The red font and asterisk indicated comparisons that were statistically significant. C-IVIG = hyperimmune anti-COVID-19 intravenous immunoglobulin, CI = confidence interval, COVID-19 = coronavirus disease 2019, CP = convalescent plasma, HS = high dosage sarilumab, IFN-β=interferon-beta, LS = low dosage sarilumab, OR = odds ratio, SOC = standard-of-care, UC-MSCs = human umbilical cord-derived mesenchymal stem cells.

In the safety outcome, data from 19 studies^[18,20,21,23,26–29,34,38–40,48,50–54,56]^were merged for analysis (Supplemental Table S2, http://links.lww.com/MD/H537). We found that colchicine (OR 2.77, 95% CI 1.03-7.42) and IFN-β/SOC (OR 18.98, 95% CI 1.70-211.84) seemed to increase the risk of TEAEs when compared with the SOC group. Moreover, IFN-β/SOC (OR 19.00, 95% CI 2.36-153.10) were associated with the increase of TEAEs when compared with placebo. We found no significant difference in TEAEs in other 15 medications for severe COVID-19 patients when compared with SOC or placebo (Fig. 5).

The figure of SUCRA showed that baricitinib had the highest cumulative probability (SUCRA: 88.3%) becoming the best intervention in TEAEs, followed by LPV/r (SUCRA: 78.8%), SOC (SUCRA: 76.0%), lenzilumab (SUCRA: 72.6%), C-IVIG (SUCRA: 68.5%), tocilizumab (SUCRA: 63.6%), placebo (SUCRA: 53.1%), ivermectin (SUCRA: 52.3%), canakinumab (SUCRA: 51.9%), mycobacterium-w (SUCRA: 50.1%), LS (SUCRA: 49.3%), mavrilimumab (SUCRA: 47.1%), ruxolitinib/SOC (SUCRA: 46.8%), CP (SUCRA: 44.5%), HS (SUCRA: 31.0%), HCQ (SUCRA: 29.5%), human umbilical cord-derived mesenchymal stem cells (SUCRA: 29.1%), colchicine (SUCRA: 14.2%), and IFN-β/SOC (SUCRA: 3.2%) (Supplemental Figure S2, http://links.lww.com/MD/H539).

### 3.6. Evaluation of inconsistency

According to the inconsistency test (Table 2), no significant inconsistency or qualitative difference was available for the ACM and TEAEs. Thus, the consistency hypothesis was accepted in this NMA.

**Table 2:** The evaluation of inconsistency for the efficacy and safety of medications among severe COVID-19 patients.

Network meta-analysis	Number of dimensions	Chi² value	*P* value
All-cause mortality	34	2.37	.668
The ratio of treatment-emergent adverse events	18	0.69	.407

COVID-19 = coronavirus disease 2019.

### 3.7. Sensitivity analysis and publication bias

**Figure 6.**
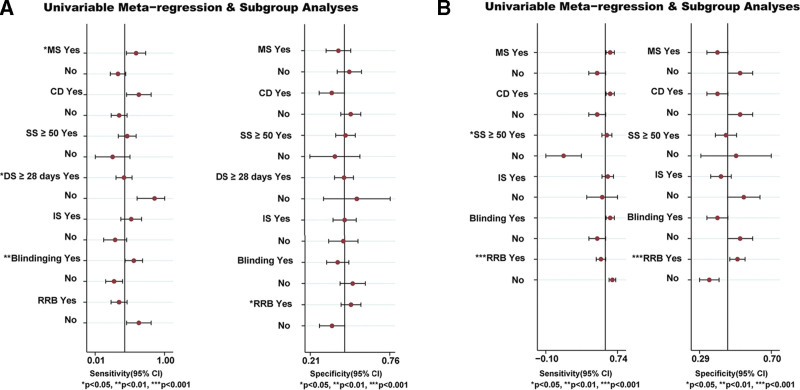
Meta-regression and sensitivity analyses for the efficacy and safety of medications in patients with severe COVID-19 infection. (A) all-cause mortality. (B) the ratio of treatment-emergent adverse events. CD = crossover design, COVID-19 = coronavirus disease 2019, DS = duration of study, IS = industry sponsorship, MS = multicenter study, RRB = risk of reported bias, SS = sample size.

We analyzed the potential sources of heterogeneity or inconsistency by using subgroup and meta-regression analyses. Univariable meta-regression and subgroup analyses indicated that there were heterogeneous sources (such as DS, blinding and RRB) for the ACM (*P* < .05) (Fig. 6A). Whilst the SS and RRB were the heterogeneity source of TEAEs based on the sensitivity analysis (*P* < .05) (Fig. 6B).

None of the funnel plots of outcomes (ACM and TEAEs) indicated a significant asymmetry (Supplemental Figure S3, http://links.lww.com/MD/H540).

## 4. Discussion

This study was based on 48 RCTs, which included 9147 severe patients randomly assigned to 35 medications or SOC or placebo. Our NMA indicated that 12 medications (i.e., ivermectin/doxycycline, C-IVIG, methylprednisolone, IFN-β/SOC, IFN-β-1b, CP, remdesivir, LPV/r, HS, IG, auxora, and imatinib) were efficacious for the therapy of severe COVID-19 infection based on the controls of SOC or placebo. For a decrease in ACM, on the one hand, C-IVIG, methylprednisolone, IFN-β/SOC, HS, remdesivir, and CP were more efficacious than SOC; on the other hand, the efficacy of ivermectin/doxycycline, C-IVIG, methylprednisolone, IFN-β/SOC, IFN-β-1b, CP, remdesivir, LPV/r, IG, auxora, and imatinib were superior to placebo in all 35 drug interventions. From this, we can infer that there is a difference in the findings of medications for severe COVID-19 patients from different control conditions in RCTs.

Curiously, our findings differed from our earlier pilot studies,^[14,60]^ which have only indicated 2 effective medications (i.e., IG and methylprednisolone) in the decreased ACM for severe COVID-19 patients. In addition, the CP group showed lower TEAEs than the placebo for severe COVID-19 infection in our earlier NMAs.^[14,60]^ These inconsistencies may be due to an increase in sample size in the updated NMA. The study extended the previous work that provided a reference for selecting the medication for patients with severe COVID-19 infection.^[61]^ The present study raises the possibility that we cannot completely discount the efficacy of other medications for severe COVID-19 infection in clinic. So far, maybe it’s because there was inadequate evidence of a benefit in ACM for them. In either case, it is noteworthy that we must fully consider its efficacy and safety when the medications are used in treating severe COVID-19 patients. Some medications (e.g., tocilizumab, otilimab, and mavrilimumab) might be a selection of combination treatment for COVID-19 infection.^[62,63]^

It is particularly worth mentioning that host factors, including age, prior-medical history, demographics, and medical facility, may be key determinants of disease severity and progression.^[64]^ Similarly, the above factors are associated with the drug treatment effect of COVID-19 infection. Several studies have reported that aging and prior-medical history (such as cancer or chronic kidney/liver/lung diseases) might be 2 prominent impact factors for medical therapy from COVID-19.^[65,66]^ Kopel *e*t al suggested that some demographics factors (such as gender, ethnic group and socioeconomic status) were associated with the therapy of COVID-19 infection.^[67,68]^ In another study in India, Sarkar et al^[69]^ reported that different strains of SARS-CoV-2 were associated with the efficacy of antiviral drugs. It has also been suggested that medical facilities may be an impact factor for the COVID-19 treatment.^[70]^ We have considered these impact factors in the overall therapy for COVID-19 patients during this NMA design. To reduce the impact of age, we included all of studies based on patient age greater than or equal to 16 years. As is well known, the design principles of RCTs are randomization, control, blinding, and repetition.^[71]^ According to the 48 RCTs data analysis of this NMA, we confirmed that age, prior-medical history, gender and racial ratios for participants were balanced between experimental and control groups (*P* > .05). In view of this, we suggest that the effects of age, prior-medical history, gender and racial ratios in the findings are small in this NMA study. This might present one of the main strengthens of this NMA. Unfortunately, the description of most studies was insufficient in the gender-specific, different strains and medical facilities for COVID-19 therapy in previous RCTs. We could not explore the efficacy of drugs treatment from the gender-specific or different strains as well as the impact from medical facilities for COVID-19 therapy.

### 4.1. Efficacy of current medications

This study supported evidence from previous observations.^[72,73]^ As we know, intravenous immunoglobulin has already been validated as an effective antiviral drug for treating COVID-19, SARS and Middle East respiratory syndrome.^[20,74,75]^ This is consistent with our results (i.e., C-IVIG, HS, and IG). Recent evidence suggested that anti-cytokine effects, inhibition of complement activation, and down-regulation of B and T cells’ functions by C-IVIG can prevent organ failure and subsequent mortality in severe COVID-19 patients.^[20]^ Our findings also confirmed that sarilumab could prevent patients with COVID-19 from progressing to death. Sarilumab is a fully human antibody against the interleukin (IL)-6 receptor, which can rapidly lower C-reactive protein and mediate COVID-19 clearance.^[76]^ Prolonged glucocorticoid treatment is associated with improved outcomes of acute respiratory distress syndrome.^[77]^ Several reports have also shown that treatment with methylprednisolone could significantly reduce the risk of death among patients with acute respiratory distress syndrome.^[78]^ This also accords with our observations, which suggested that methylprednisolone was associated with decreased ACM in severe COVID-19 patients.

The SUCRA indicated that ivermectin/doxycycline was the highest ranked intervention with a SUCRA of 0.821. Previous studies showed that ivermectin or doxycycline is effective in treating COVID-19 patients.^[79,80]^ We attribute this to a couple of reasons. First, ivermectin or doxycycline might possess antiviral as well as immunomodulatory activity.^[79,80]^ Second, they might have anti-inflammatory and immunomodulatory agents and can curb over-reacting innate and cellular immune responses.^[81]^ One possible implication may be that the ivermectin/doxycycline is an excellent selection for treating severe COVID-19 patients. Of note, based on the result of meta-regression analysis on the heterogeneity (such as DS, blinding, and RRB), the present result may need further verification. Thus, statistical indications of clinical superiority in this network analysis required careful interpretation.

Interestingly, we found that small-molecule protein kinase inhibitors (i.e., auxora and imatinib) were efficacious for the therapy of severe COVID-19 infection. Recent research has established that a calcium release-activated calcium channel inhibitor, such as auxora, can reduce the occurrence of COVID-19 death due to blocking the release of multiple pro-inflammatory cytokines, including IL-6. Additionally, evidence showed that imatinib might play its potentially antiviral and beneficial immunomodulatory role in severe COVID-19 patients.^[82]^ These results further support those of previous studies.^[25,83]^ Collectively, the present findings indicate that the small-molecule protein kinase inhibitors provide a new, and perhaps superior, avenue for the severe COVID-19 treatment. In addition, our results may provide a robust strategy for clinical combination therapy among severe COVID-19 patients.

We have demonstrated that IFN-β/SOC, IFN-β-1b, remdesivir, LPV/r and CP were associated with a reduction of ACM in severe COVID-19 infection. Several lines of evidence suggested that IFN deficiency was a hallmark of severe COVID-19.^[84]^ IFN might treat severe COVID-19 infection through adapted anti-inflammatory therapies that target IL-6 or TNF-β.^[84,85]^ Data from prior studies suggested that remdesivir was a broad-spectrum antiviral activity against RNA viruses, which could improve the survival of COVID-19 infection due to inhibiting viral replication.^[86]^ Recent studies reported that LPV/r displayed inhibitory activities against SARS-CoV-2 main protease and inhibited SARS-CoV-2 replication in Vero E6 cells.^[87]^ Similarly to other antibodies therapy, CP can prevent the death of severe COVID-19 patients because of alleviating the inflammation and overreaction of the immune system through antibodies.^[88]^ These findings further support the idea of our NMA study.

Conversely, no significant difference was found in other 21 medications (e.g., HCQ, α-Lipoic acid, hydrocortisone, otilimab, and mavrilimumab) for the ACM of severe COVID-19 infection when compared with SOC or placebo. This finding was contrary to previous studies which have suggested that some antiviral drugs (hydrocortisone, mavrilimumab, etc) were associated with decreased ACM in patients with COVID-19.^[29,32,89,90]^ It is difficult to explain this result, but it might be related to the difference of participants’ selection in differential studies. For instance, the findings of this NMA were based on a larger sample size (i.e., more participants were included). Correspondingly, most of the previous studies presented a smaller sample size.^[29,32]^ The present study differed from earlier studies,^[90,91]^ which selected subjects with all infection levels (i.e., mild, moderate and severe infections). Furthermore, prior studies were inadequate for the analyses stratified by different infection levels (i.e., non-severe and severe infection) in medications of COVID-19.^[29,32,89,90]^ Prior studies suggested that COVID-19 patients at different infection levels often led to different outcomes of treatment.^[92]^ Another possible explanation for this was that we compared the efficacy and safety of SOC, which existed the bias due to the differential SOC of every country (i.e., the SOC is not standardized) except for the reasons given above.^[21,23,45]^ The present study raised the possibility that our findings might be beneficial to guiding the selection of drug interventions for clinicians in severe COVID-19 patients.

### 4.2. Safety of current medications

In terms of safety, we summarized the TEAEs. We found that colchicine and IFN-β/SOC were only associated with the TEAEs of severe COVID-19 patients in this study. Recent meta-analysis studies and large-scale RCTs^[45,46,92]^ seemed to be consistent with our findings, which identified most pharmacological treatments had a good safety in treating severe COVID-19. However, as mentioned in the present study, colchicine and IFN-β/SOC should be chosen cautiously in treating severe COVID-19 patients based on safety. Colchicine might increase the TEAEs in treating patients with severe COVID-19 infection. It is difficult to explain this result, but it might be related to the toxicity (e.g., gastrointestinal mucosal damage) in the case of colchicine treatment.^[93]^ In addition, it is possible that the use of IFN-β in combination with SOC was associated with increased TEAEs in treating severe COVID-19, which should be weighed in all future IFN-β studies.^[94]^

In summary, clinicians might need to select treatment regimens based on the ranks of efficacy and safety (i.e., SUCRA) when medications are used in treating severe COVID-19 patients. Additionally, it should be reminded that further studies, which reduce the effects of SS and RRB, will need to be undertaken based on the result of sensitivity analysis.

Some limitations constrained this study. Firstly, included studies might be small in this NMA, which should be considered when interpreting the findings. Secondly, the published data we extracted included only 2 types of outcomes, some important outcomes such as discharge ratio, clinical improvement ratio, and the ratio of virological cure, were not analyzed. Thirdly, although we did our best to include all available RCTs, we cannot eliminate the possibility of missing data. Fourthly, some nodes in our network included only a few trials. The sample size of actual head-to-head trials was very small. Hence, comparative efficacy and safety between interventions was frequently based on indirect comparisons. Finally, the sensitivity analysis showed that there were several heterogeneity sources, which may conceal or exaggerate the effect size of this network analysis. Further large-scale RCTs, which control these confounding factors, will need to be undertaken to verify our findings. Though there are still many shortcomings in our research, it is certain that the prevention and therapy of COVID-19 is set to change for the better in the future.

## 5. Conclusions

In conclusion, this NMA demonstrated that ivermectin/doxycycline, C-IVIG, methylprednisolone, IFN-β/SOC, IFN-β-1b, CP, remdesivir, LPV/r, IG, HS, auxora, and imatinib were effective for treating severe COVID-19 patients. There may be a difference in the findings of medications for severe COVID-19 patients from different control conditions (i.e., placebo and SOC) in RCTs. We found that most medications were safe in treating severe COVID-19. The present NMA reported uncertain estimates on the efficacy and safety of medications in the severe COVID-19 treatment. Maybe it’s because there was inadequate evidence of a reduction in ACM and the absence of TEAEs. However, this study had 2 strengths. One was that a comprehensive meta-analysis strategy was used to reduce the risk of publication bias. The other was that the SUCRA was used to assess possibly the best intervention.

Despite these limitations, to date, the present findings might represent the most comprehensive meta-analysis of the available evidence for severe COVID-19 infection. Future guidelines and decision-making treatment plan should consider these results for the severe COVID-19 treatment. Importantly, the government, academia and researchers should collaborate to develop more large-scale RCTs studies and further estimate the efficacy and safety of treatment interventions on mortality, virological and clinical outcomes for different levels of infection with COVID-19.

## Author contributions

**Conceptualization:** Cheng Qing Lin, Zhao Gang.

**Data curation:** Chen Jun Fang, Jia Qing Jun, Fang Zi Jian.

**Formal analysis:** Cheng Qing Lin, Zhao Gang.

**Funding acquisition:** Cheng Qing Lin.

**Methodology:** Cheng Qing Lin, Chen Jun Fang.

**Supervision:** Jia Qing Jun, Fang Zi Jian.

**Writing – original draft:** Cheng Qing Lin.

**Writing – review & editing:** Cheng Qing Lin, Zhao Gang.

## Acknowledgments

We acknowledge with gratitude all study authors who responded to our data requests. We thank many researchers who sent information for our previous reviews on which this report was built. This work is supported by the Basic Public Welfare Research Project of Zhejiang Province (grant number: LGF21H260007), the Medical Science and Technology Project of Zhejiang Province (grant numbers: 2020PY064 and 2021PY065), and the Key Medical Discipline construction project (Disinfection and Vector Control) of Hangzhou.

## Supplementary Material


