# Supplementary material for: Comparative efficacy and safety of pharmacological interventions for severe COVID-19 patients: An updated network meta-analysis of 48 randomized controlled trials
Source: Medicine (Baltimore). 2022 Oct 14;101(41):e30998. doi: 10.1097/MD.0000000000030998 (PMC9575403; doi:10.1097/MD.0000000000030998)
Supplement: Supplementary file 1 [file medi-101-e30998-s001.pdf]

Appendix 1

Full search strategies:

We used the search terms "coronavirus disease*" OR "COVID*" OR "coronavirus disease 19*" OR "COVID-19*" OR "SARS-CoV-2" OR "treatment*" OR "therapy*" OR "hydroxychloroquine*" OR "chloroquine*" OR "ivermectin*" OR "avifavir*" OR "doxycycline*" OR "sarilumab*" OR "colchicine*" OR "interferon*" OR "lopinavir/ritonavir*" OR "convalescent plasma*" OR "arbidol*" OR "remdesivir*" OR "standard of care*" OR "α-Lipoic acid*" OR "monoclonal antibody*" OR "auxora*" OR "tocilizumab*" OR "hormone*" OR "otilimab*" OR "immunoglobulin gamma *" combined with a list of all included antiviral drugs.
