# Supplementary material for: Comparative efficacy and safety of pharmacological interventions for severe COVID-19 patients: An updated network meta-analysis of 48 randomized controlled trials
Source: Medicine (Baltimore). 2022 Oct 14;101(41):e30998. doi: 10.1097/MD.0000000000030998 (PMC9575403; doi:10.1097/MD.0000000000030998)
Supplement: Supplementary file 2 [file medi-101-e30998-s002.pdf]

Appendix 2

Full inclusion and exclusion criteria

Studies were ultimately included if they (a) were a randomized controlled trial (RCT); (b) were the RCTs of at least 1 week's duration; (c) were severe COVID-19 patients aged 16 years and older; (d) reported COVID-19 related therapy methods as a predictor of clinical outcomes (efficacy or safety), including the proportion of all-cause mortality (ACM), and treatment-emergent adverse events (TEAEs); and (e) reported any of the following statistics: ACM, the ratio of TEAEs and sample sizes, or other statistics that could be converted into a standardized effect size.

Studies were excluded if they (a) were wrong study design or population; (b) were duplicated research or not full-text articles; (c) had no outcomes/drugs of interest; (d) reported the publication types of non-clinical studies, non-randomized controlled trial, review articles, commentaries, guidelines, and meta-analysis; (e) no primary or missing data existed after contacting authors; or (f) had considerable heterogeneity of studies' groups.
