# Supplementary material for: Comparative efficacy and safety of pharmacological interventions for severe COVID-19 patients: An updated network meta-analysis of 48 randomized controlled trials
Source: Medicine (Baltimore). 2022 Oct 14;101(41):e30998. doi: 10.1097/MD.0000000000030998 (PMC9575403; doi:10.1097/MD.0000000000030998)
Supplement: Supplementary file 3 [file medi-101-e30998-s003.pdf]

Appendix 3

The network meta-analysis program of STATA software for all-cause mortality in patients with COVID-19

network setup r n, stud(study) trt(t) num ref(SOC)

network map, improve

network meta i

network meta consistency

network sidesplit all, tau

netweight _y _stderr _t1 _t2, color(navy) symbol(circle) title(Contribution graph for COVID-19) bargraph(by blind mean)

network rank min, all zero reps(5000) gen(prob)

network rank min, zero all reps(5000) gen(pred_prob) predict

sucra prob, lab(SOC ALA Auxora Azithromycin/SOC Baricitinib C-IVIG CP CP/SOC Canakinumab Chloroquine Chloroquine/hydroxychloroquine Colchicine HDIVC HS Hydrocortisone Hydroxychloroquine IFN-β-1b IG Imatinib Interferon-beta/SOC Ivermectin Ivermectin/doxycycline LS Lenzilumab Lopinavir/Ritonavir Mavrilimumab Methylprednisolone Mycobacterium-w N-acetylcysteine Otilimab Placebo Remdesivir Remdesivir/SOC Ruxolitinib/SOC Tocilizumab)*

netleague, lab(SOC ALA Auxora ASOC Baricitinib CIVIG CP CSOC Canakinumab Chloroquine ChHCQ Colchicine HDIVC HS Hydrocortisone Hydroxychloroquine IFNβb IG Imatinib IFNSOC Ivermectin Ivermectind LS Lenzilumab LopinavirR Mavrilimumab Methylprednisolone Mycobacteriumw Nacetylcysteine Otilimab Placebo Remdesivir RemdS RuxolitinibS Tocilizumab) sort(Ivermectind RuxolitinibS ALA CIVIG Methylprednisolone IG IFNSOC IFNβb Mavrilimumab Auxora Baricitinib Imatinib CP Remdesivir LopinavirR

HDIVC CSOC Hydrocortisone Lenzilumab Canakinumab Colchicine RemdS Mycobacteriumw Ivermectin SOC ASOC Otilimab Nacetylcysteine LS Tocilizumab Placebo Hydroxychloroquine Chloroquine HS ChHCQ) eform

network convert pairs

netfunnel _y _stderr _t1 _t2 , random bycomp add(lfit _stderr _ES_CEN) noalpha

ifplot _y _stderr _t1 _t2 study, tau2(loop)

midas tp fp fn tn,reg(ms cd ss ds is blinding rrb)

The network meta-analysis program of STATA software for the rate of treatment-emergent adverse events in patients with COVID-19

network setup r n, stud(study) trt(t) num ref(SOC)

network map, improve

network meta i

network meta c, fixed

network sidesplit all, tau

network rank min, all zero reps(5000) gen(prob)

netweight _y _stderr _t1 _t2, color(navy) symbol(circle) title(Contribution graph for COVID-19) fixed bargraph(by blind mean)

network rank min,zero all reps(5000) gen(pred_prob) predict

sucra prob, lab(SOC Baricitinib C-IVIG CP Canakinumab Colchicine HS Hydroxychloroquine Interferon-beta/SOC Ivermectin LPV/r LS Lenzilumab Mavrilimumab Mycobacterium-w Placebo Ruxolitinib/SOC Tocilizumab UC-MSCs)*

```
netleague, lab(SOC Baricitinib CIVIG CP Canakinumab Colchicine HS Hydroxychloroquine InterferonS Ivermectin LPVr LS Lenzilumab Mavrilimumab Mycobacteriumw Placebo RSOC Tocilizumab UCMSCs) sort(Baricitinib Lenzilumab LPVr Placebo SOC Canakinumab LS CIVIG Mavrilimumab Mycobacteriumw CP Ivermectin Tocilizumab HS RSOC Hydroxychloroquine UCMSCs Colchicine InterferonS) eform

network convert pairs

netfunnel _y _stderr _t1 _t2 , random bycomp add(lfit _stderr _ES_CEN) noalpha

ifplot _y _stderr _t1 _t2 study, tau2(loop)

midas tp fp fn tn,reg(ms cd ss ds is blinding rrb)
```
