# Supplementary material for: Comparative efficacy and safety of pharmacological interventions for severe COVID-19 patients: An updated network meta-analysis of 48 randomized controlled trials
Source: Medicine (Baltimore). 2022 Oct 14;101(41):e30998. doi: 10.1097/MD.0000000000030998 (PMC9575403; doi:10.1097/MD.0000000000030998)
Supplement: Supplementary file 4 [file medi-101-e30998-s004.pdf]

Table S1
Included studies of network meta-analysis for all-cause mortality in patients with COVID-19.

Study	Intervention and control groups	Events	n	All-cause mortality (%)
Absalón-Aguilar A *et al.* 2021	Colchicine	4	56	7.14
Absalón-Aguilar A *et al.* 2021	Placebo	6	60	10.00
Ader F *et al.* 2022	Remdesivir/SOC	19	161	11.80
Ader F *et al.* 2022	SOC	22	167	13.17
Ali S *et al.* 2021	C-IVIG	10	40	25.00
Ali S *et al.* 2021	SOC	6	10	60.00
AlQahtani M *et al.* 2021	Convalescent plasma	1	20	5.00
AlQahtani M *et al.* 2021	SOC	2	20	10.00
AlShehry N *et al.* 2021	Convalescent plasma	10	40	25.00
AlShehry N *et al.* 2021	SOC	46	124	37.10
Aman J *et al.* 2021	Imatinib	15	197	7.61
Aman J *et al.* 2021	Placebo	27	188	14.36
Avendano Sola C *et al.* 2020	Convalescent plasma	0	38	0.00
Avendano Sola C *et al.* 2020	SOC	4	43	9.30
Beltran-Gonzalez JL *et al.* 2021	Hydroxychloroquine	2	33	6.06
Beltran-Gonzalez JL *et al.* 2021	Ivermectin	5	36	13.89
Beltran-Gonzalez JL *et al.* 2021	Placebo	6	37	16.22
Bruen C *et al.* 2022	Auxora	10	130	7.69
Bruen C *et al.* 2022	Placebo	23	131	17.56
Cao B *et al.* 2020	Lopinavir/Ritonavir	16	99	16.16
Cao B *et al.* 2020	SOC	25	100	25.00
Cao Y *et al.* 2020	Ruxolitinib/SOC	0	20	0.00
Cao Y *et al.* 2020	SOC	3	21	14.29
Caricchio R *et al.* 2021	Canakinumab	11	223	4.93
Caricchio R *et al.* 2021	Placebo	16	222	7.21
Cremer PC *et al.* 2021	Mavrilimumab	1	21	4.76
Cremer PC *et al.* 2021	Placebo	3	19	15.79
Davoudi-Monfared E *et al.* 2020	Interferon-beta/SOC	8	42	19.05
Davoudi-Monfared E *et al.* 2020	SOC	17	39	43.59
de Alencar JCG *et al.* 2021	N-acetylcysteine	9	67	13.43
de Alencar JCG *et al.* 2021	Placebo	9	68	13.24
Dequin PF *et al.* 2020	Hydrocortisone	11	76	14.47
Dequin PF *et al.* 2020	Placebo	20	73	27.40
Edalatifard M *et al.* 2020	Methylprednisolone	2	34	5.88
Edalatifard M *et al.* 2020	SOC	12	28	42.86
Ely EW *et al.* 2022	Baricitinib	20	51	39.22
Ely EW *et al.* 2022	Placebo	29	50	58.00
Furtado RHM *et al.* 2020	Azithromycin/SOC	90	214	42.06
Furtado RHM *et al.* 2020	SOC	73	183	39.89
Gharebaghi N *et al.* 2020	Immunoglobulin gamma	6	30	20.00
Gharebaghi N *et al.* 2020	Placebo	14	29	48.28
Hashim HA *et al.* 2021	Ivermectin/doxycycline	0	11	0.00
Hashim HA *et al.* 2021	SOC	6	22	27.27
Hernandez-Cardenas C *et al.* 2021	Hydroxychloroquine	40	106	37.74
Hernandez-Cardenas C *et al.* 2021	Placebo	44	108	40.74
Lescure FX *et al.* 2021	Low dosage sarilumab	17	159	10.69
Lescure FX *et al.* 2021	High dosage sarilumab	18	173	10.40
Lescure FX *et al.* 2021	Placebo	9	84	10.71
Li L *et al.*2020	Convalescent plasma/SOC	8	52	15.38

Li L *et al.*2020	SOC	12	51	23.53
Libster R *et al.*2021	Convalescent plasma	2	80	2.50
Libster R *et al.*2021	Placebo	4	80	5.00
Miller J *et al.*2020	Auxora	2	20	10.00
Miller J *et al.*2020	SOC	2	10	20.00
Munch MW *et al.*2021	Hydrocortisone	6	16	37.50
Munch MW *et al.*2021	Placebo	2	14	14.29
Olender SA *et al.*2020	Remdesivir	24	312	7.69
Olender SA *et al.*2020	SOC	102	818	12.47
Patel J *et al.*2021	Otilimab	93	403	23.08
Patel J *et al.*2021	Placebo	97	403	24.07
Rahmani H *et al.*2021	Interferon-beta-1b	2	33	6.06
Rahmani H *et al.*2021	SOC	6	33	18.18
Rasheed AM *et al.*2020	Convalescent plasma	1	21	4.76
Rasheed AM *et al.*2020	SOC	8	28	28.57
Rea-Neto A *et al.*2021	Chloroquine/hydroxychloroquine	16	53	30.19
Rea-Neto A *et al.*2021	Chloroquine	7	24	29.17
Rea-Neto A *et al.*2021	Hydroxychloroquine	8	29	27.59
Rea-Neto A *et al.*2021	SOC	10	52	19.23
Rosas IO *et al.*2021	Tocilizumab	58	294	19.73
Rosas IO *et al.*2021	Placebo	28	144	19.44
Sehgal IS *et al.*2021	Mycobacterium-w	4	20	20.00
Sehgal IS *et al.*2021	Placebo	5	22	22.73
Simonovich VA *et al.*2020	Convalescent plasma	0	228	0.00
Simonovich VA *et al.*2020	Placebo	0	105	0.00
Sivapalasingam S *et al.*2021	Low dosage sarilumab	60	242	24.79
Sivapalasingam S *et al.*2021	High dosage sarilumab	103	338	30.47
Sivapalasingam S *et al.*2021	Placebo	43	170	25.29
Solanich X *et al.*2021	Methylprednisolone	4	27	14.81
Solanich X *et al.*2021	SOC	6	28	21.43
Temesgen Z *et al.*2021	Lenzilumab	23	236	9.75
Temesgen Z *et al.*2021	Placebo	34	243	13.99
Veiga VC *et al.*2021	Tocilizumab	14	65	21.54
Veiga VC *et al.*2021	SOC	6	64	9.38
Zhang J *et al.*2021	High-dose intravenous vitamin C	6	27	22.22
Zhang J *et al.*2021	Placebo	10	29	34.48
Zhong M *et al.*2020	α -Lipoic acid	3	8	37.50
Zhong M *et al.*2020	Placebo	7	9	77.78

COVID-19= coronavirus disease 2019, C-IVIG= hyperimmune anti-COVID-19 intravenous immunoglobulin, SOC= standard of care.
