# Supplementary material for: Comparative efficacy and safety of pharmacological interventions for severe COVID-19 patients: An updated network meta-analysis of 48 randomized controlled trials
Source: Medicine (Baltimore). 2022 Oct 14;101(41):e30998. doi: 10.1097/MD.0000000000030998 (PMC9575403; doi:10.1097/MD.0000000000030998)
Supplement: Supplementary file 5 [file medi-101-e30998-s005.pdf]

Table S2

Included studies of network meta-analysis for treatment-emergent adverse events in patients with severe COVID-19.

Study	Intervention and control groups	Events	n	The ratio of treatment-emergent adverse (%)
Absalón-Aguilar A *et al.* 2021	Colchicine	15	56	26.79
Absalón-Aguilar A *et al.* 2021	Placebo	7	60	11.67
Ali S *et al.* 2021	C-IVIG	28	40	70.00
Ali S *et al.* 2021	SOC	7	10	70.00
AlQahtani M *et al.* 2021	Convalescent plasma	2	20	10.00
AlQahtani M *et al.* 2021	SOC	0	20	0.00
Avendano Sola C *et al.* 2020	Convalescent plasma	6	38	15.79
Avendano Sola C *et al.* 2020	SOC	7	43	16.28
Cao B *et al.* 2020	Lopinavir/Ritonavir	46	99	46.46
Cao B *et al.* 2020	SOC	49	100	49.00
Cao Y *et al.* 2020	Ruxolitinib/SOC	14	20	70.00
Cao Y *et al.* 2020	SOC	12	21	57.14
Caricchio R *et al.* 2021	Canakinumab	122	225	54.22
Caricchio R *et al.* 2021	Placebo	120	223	53.81
Cremer PC *et al.* 2021	Mavrilimumab	5	21	23.81
Cremer PC *et al.* 2021	Placebo	4	19	21.05
Ely EW *et al.* 2022	Baricitinib	44	50	88.00
Ely EW *et al.* 2022	Placebo	47	49	95.92
Hernandez-Cardenas C *et al.* 2021	Hydroxychloroquine	15	106	14.15
Hernandez-Cardenas C *et al.* 2021	Placebo	10	108	9.26
Krolewiecki A *et al.* 2021	Ivermectin	13	30	43.33
Krolewiecki A *et al.* 2021	SOC	5	15	33.33
Lescure FX *et al.* 2021	Placebo	55	84	65.48
Lescure FX *et al.* 2021	Low dosage sarilumab	103	159	64.78
Lescure FX *et al.* 2021	High dosage sarilumab	121	173	69.94
Rasheed AM *et al.* 2020	Convalescent plasma	1	21	4.76
Rosas IO *et al.* 2021	Tocilizumab	228	295	77.29
Rosas IO *et al.* 2021	Placebo	116	143	81.12
Sehgal IS *et al.* 2021	Mycobacterium-w	0	20	0.00
Sehgal IS *et al.* 2021	Placebo	0	22	0.00
Shi L *et al.* 2021	UC-MSCs	37	65	56.92
Shi L *et al.* 2021	Placebo	21	35	60.00
Simonovich VA *et al.* 2020	Convalescent plasma	153	228	67.11
Simonovich VA *et al.* 2020	Placebo	66	105	62.86
Sivapalasingam S *et al.* 2021	Low dosage sarilumab	19	50	38.00
Sivapalasingam S *et al.* 2021	High dosage sarilumab	25	51	49.02
Sivapalasingam S *et al.* 2021	Placebo	7	25	28.00
Temesgen Z *et al.* 2021	Lenzilumab	68	255	26.67

Temesgen Z *et al*. 2021	Placebo	84	257	32.68
Veiga VC *et al.* 2021	Tocilizumab	29	67	43.28
Veiga VC *et al.* 2021	SOC	21	62	33.87
Davoudi Monfared E *et al.* 2020	Interferon-beta/SOC	14	42	33.33
Davoudi Monfared E *et al.* 2020	SOC	1	39	2.56

COVID-19= coronavirus disease 2019, C-IVIG= hyperimmune anti-COVID-19 intravenous immunoglobulin, SOC= standard of care, UC-MSCs= human umbilical cord-derived mesenchymal stem cells.
