# Supplementary material for: Comparative efficacy and safety of pharmacological interventions for severe COVID-19 patients: An updated network meta-analysis of 48 randomized controlled trials
Source: Medicine (Baltimore). 2022 Oct 14;101(41):e30998. doi: 10.1097/MD.0000000000030998 (PMC9575403; doi:10.1097/MD.0000000000030998)
Supplement: Supplementary file 6 [file medi-101-e30998-s006.pdf]

Supplemental Figure S1. The ranking for the efficacy of medications based on cumulative probability plots and surface under the cumulative ranking area among severe COVID-19 patients.

COVID-19= coronavirus disease 2019, SOC= standard-of-care, ALA= α-Lipoic acid, IFN-β= interferon-beta, CP= convalescent plasma, C-IVIG= hyperimmune anti-COVID-19 intravenous immunoglobulin, IG= immunoglobulin gamma, HDIVC= high-dose intravenous vitamin C, HS= high dosage sarilumab, LS= low dosage sarilumab.

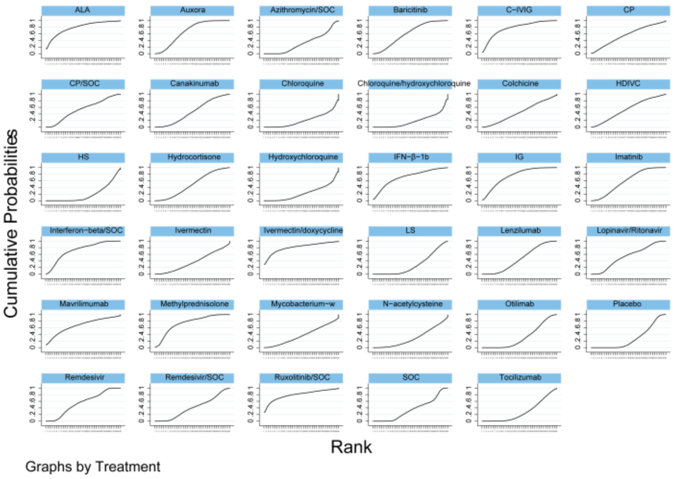

Treatment Relative Ranking of Model 1

Treatment	SUCRA	PrBest	MeanRank
SOC	35.5	0.0	22.9
ALA	79.1	15.2	8.1
Auxora	67.3	0.2	12.1
Azithromycin/SOC	32.4	0.0	24.0
Baricitinib	60.7	0.2	14.4
C-IVIG	77.8	5.2	8.6
CP	56.5	1.4	15.8
CP/SOC	53.5	0.0	16.8
Canakinumab	47.0	0.0	19.0
Chloroquine	21.3	0.0	27.8
Chloroquine/hydroxychloroquine	18.9	0.0	28.6
Colchicine	45.2	0.1	19.6
HDIVC	54.0	0.4	16.6
HS	19.8	0.0	28.3
Hydrocortisone	49.1	0.1	18.3
Hydroxychloroquine	22.9	0.0	27.2
IFN-β-1b	71.3	4.2	10.8
IG	75.0	2.7	9.5
Imatinib	59.4	0.1	14.8
Interferon-beta/SOC	72.7	1.1	10.3
Ivermectin	38.4	0.1	21.9
Ivermectin/doxycycline	82.1	30.1	7.1
LS	30.2	0.0	24.7
Lenzilumab	47.5	0.0	18.8
Lopinavir/Ritonavir	54.8	0.0	16.4
Mavrilimumab	68.5	9.2	11.7
Methylprednisolone	75.5	1.9	9.3
Mycobacterium-w	38.5	0.3	21.9
N-acetylcysteine	30.8	0.0	24.5
Otilimab	31.9	0.0	24.1
Placebo	28.6	0.0	25.3
Remdesivir	55.0	0.0	16.3
Remdesivir/SOC	40.6	0.0	21.2
Ruxolitinib/SOC	79.6	27.4	7.9
Tocilizumab	28.6	0.0	25.3
