# Supplementary material for: Comparative efficacy and safety of pharmacological interventions for severe COVID-19 patients: An updated network meta-analysis of 48 randomized controlled trials
Source: Medicine (Baltimore). 2022 Oct 14;101(41):e30998. doi: 10.1097/MD.0000000000030998 (PMC9575403; doi:10.1097/MD.0000000000030998)
Supplement: Supplementary file 7 [file medi-101-e30998-s007.pdf]

Supplemental Figure S2. The ranking for the safety of medications based on cumulative probability plots and surface under the cumulative ranking area among severe COVID-19 patients.
COVID-19= coronavirus disease 2019, SOC= standard-of-care, CP= convalescent plasma, C-IVIG= hyperimmune anti-COVID-19 intravenous immunoglobulin, LPV/r= lopinavir/ritonavir, HS= high dosage sarilumab, LS= low dosage sarilumab, UC-MSCs= human umbilical cord-derived mesenchymal stem cells.

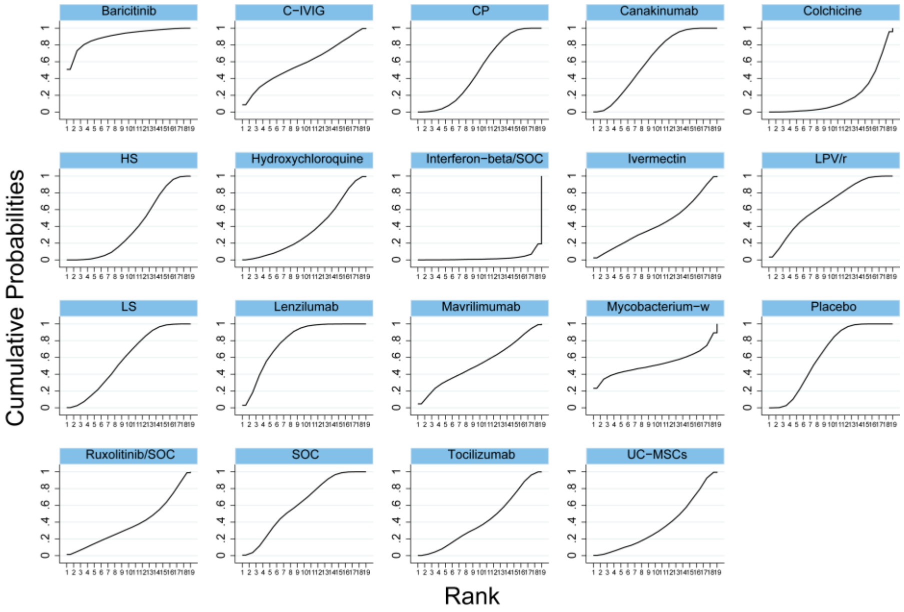

Treatment Relative Ranking of Model 1

Treatment	SUCRA	PrBest	MeanRank
SOC	76.0	1.0	5.3
Baricitinib	88.3	46.2	3.1
C-IVIG	68.5	13.3	6.7
CP	44.5	0.0	11.0
Canakinumab	51.9	0.0	9.7
Colchicine	14.2	0.0	16.4
HS	31.0	0.0	13.4
Hydroxychloroquine	29.5	0.1	13.7
Interferon-beta/SOC	3.2	0.0	18.4
Ivermectin	52.3	3.6	9.6
LPV/r	78.8	6.4	4.8
LS	49.3	0.1	10.1
Lenzilumab	72.6	1.1	5.9
Mavrilimumab	47.1	3.4	10.5
Mycobacterium-w	50.1	22.2	10.0
Placebo	53.1	0.0	9.4
Ruxolitinib/SOC	46.8	2.4	10.6
Tocilizumab	63.6	0.1	7.5
UC-MSCs	29.1	0.2	13.8
