# Supplementary material for: Comparative efficacy and safety of pharmacological interventions for severe COVID-19 patients: An updated network meta-analysis of 48 randomized controlled trials
Source: Medicine (Baltimore). 2022 Oct 14;101(41):e30998. doi: 10.1097/MD.0000000000030998 (PMC9575403; doi:10.1097/MD.0000000000030998)
Supplement: Supplementary file 8 [file medi-101-e30998-s008.pdf]

Supplemental Figure S3. Funnel plots of publication bias for the efficacy and safety of medications in patients with severe COVID-19 infection. **A**. all-cause mortality (01= α -Lipoic acid, 02=Auxora, 03=Azithromycin/Standard-of-care, 04= Baricitinib, 05=Hyperimmune anti-COVID-19 intravenous immunoglobulin, 06=Convalescent plasma, 07= Convalescent plasma/Standard-of-care, 08= Canakinumab, 09= Chloroquine, 10=Chloroquine/hydroxychloroquine, 11= Colchicine,12= High-dose intravenous vitamin C, 13=High dosage sarilumab, 14=Hydrocortisone, 15=Hydroxychloroquine, 16=Interferon beta-1b, 17=Immunoglobulin gamma, 18=Imatinib, 19= Interferon beta/Standard-of-care, 20=Ivermectin, 21=Ivermectin/doxycycline, 22=Low dosage sarilumab, 23=Lenzilumab, 24=Lopinavir/Ritonavir, 25=Mavrilimumab, 26=Methylprednisolone, 27=Mycobacterium-w, 28=N-acetylcysteine, 29=Otilimab, 30=Placebo, 31=Remdesivir, 32= Remdesivir/SOC, 33=Ruxolitinib/Standard-of-care, 34=Standard-of-care, 35=Tocilizumab). **B**. the ratio of treatment-emergent adverse events (01= Baricitinib, 02=Hyperimmune anti-COVID-19 intravenous immunoglobulin, 03=Convalescent plasma, 04=Canakinumab, 05= Colchicine, 06=High dosage sarilumab, 07=Hydroxychloroquine, 08= Interferon-beta/SOC, 09=Ivermectin, 10=Lopinavir/Ritonavir, 11=Low dosage sarilumab, 12=Lenzilumab, 13=Mavrilimumab, 14=Mycobacterium-w, 15=Placebo, 16= Ruxolitinib/Standard-of-care, 17=Standard-of-care, 18=Tocilizumab, 19=human umbilical cord-derived mesenchymal stem cells).
COVID-19= coronavirus disease 2019.

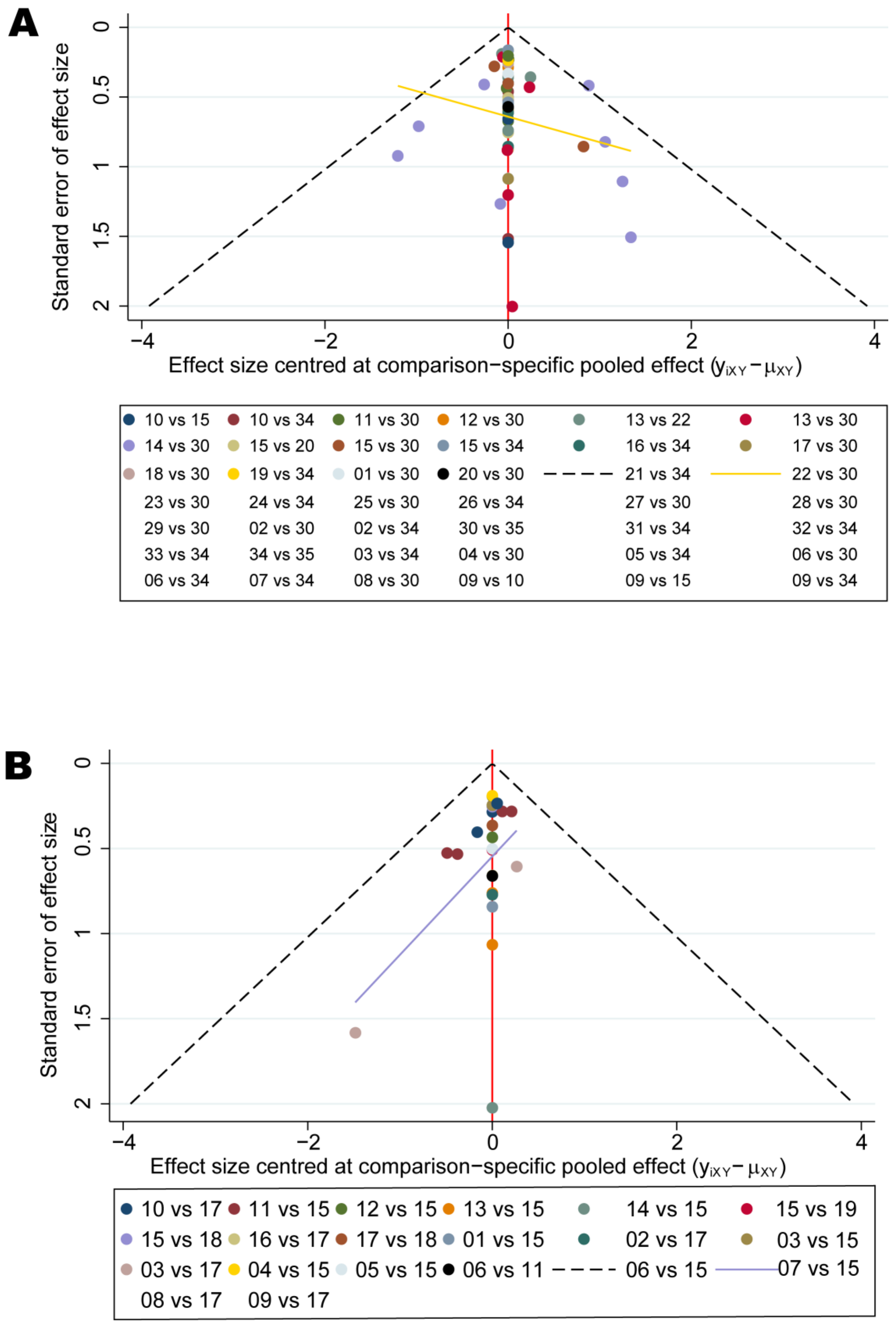
A
Standard error of effect size
0
0.5
1
1.5
2
−4
−2
0
2
4
Effect size centred at comparison−specific pooled effect ($y_{iXY} - \mu_{XY}$)
10 vs 15
10 vs 34
11 vs 30
12 vs 30
13 vs 22
13 vs 30
14 vs 30
15 vs 20
15 vs 30
15 vs 34
16 vs 34
17 vs 30
18 vs 30
19 vs 34
01 vs 30
20 vs 30
21 vs 34
22 vs 30
23 vs 30
24 vs 34
25 vs 30
26 vs 34
27 vs 30
28 vs 30
29 vs 30
02 vs 30
02 vs 34
30 vs 35
31 vs 34
32 vs 34
33 vs 34
34 vs 35
03 vs 34
04 vs 30
05 vs 34
06 vs 30
06 vs 34
07 vs 34
08 vs 30
09 vs 10
09 vs 15
09 vs 34
B
Standard error of effect size
0
0.5
1
1.5
2
−4
−2
0
2
4
Effect size centred at comparison−specific pooled effect ($y_{iXY} - \mu_{XY}$)
10 vs 17
11 vs 15
12 vs 15
13 vs 15
14 vs 15
15 vs 19
15 vs 18
16 vs 17
17 vs 18
01 vs 15
02 vs 17
03 vs 15
03 vs 17
04 vs 15
05 vs 15
06 vs 11
06 vs 15
07 vs 15
08 vs 17
09 vs 17
